# Composition and Workability of Plastic Fractions Recovered from Commingled Waste Discarded by a Composting Plant

**DOI:** 10.3390/polym15071690

**Published:** 2023-03-28

**Authors:** Claudio Badini, Oxana Ostrovskaya, Giulia Bernagozzi, Andrea Artusio

**Affiliations:** 1 Department of Applied Science and Technology, Politecnico di Torino, Corso Duca degli Abruzzi 24, 10129 Torino, Italy; 2 Department of Applied Science and Technology, Branch in Alessandria of Politecnico di Torino, Viale Teresa Michel 5, 15121 Alessandria, Italy; 3 Gestione Ambientale Integrata dell’Astigiano S.p.A. (GAIA), Via A.Brofferio 48, 14100 Asti, Italy

**Keywords:** plastic recovery, organic fraction of municipal solid waste (OFMSW), composting plant, NIR separator, recycling, tensile features

## Abstract

This paper deals with the recovery of plastic fractions from waste discarded by an industrial composting plant that processes the organic fraction of municipal solid waste. Polymeric fractions (PE, PP and PET) were sorted from this discarded waste using a NIR separator. The polymeric fractions were then washed to remove residual contaminants and characterized with the aim of assessing their composition. A process of pelletizing and injection molding suitable for producing specimens made of 100% of these recovered materials was set up. The tensile strength and stiffness, as well as the microstructure of the recycled plastics, were investigated. The mechanical features of samples fully made of recycled PE and PP were like those characteristic of virgin polymers. Samples made of PET did not show completely satisfactory properties, as they displayed rather poor elastic modulus and ductility.

## 1. Introduction

The effective management of Municipal Solid Waste (MSW) has become one of the most important issues in the world. In the last twenty years, the total amount of MSW has constantly grown due to urbanization, population growth and industrialization. Several billion tons of MSW are generated annually and the amount of MSW is expected to increase every year, which will result in an increase of 70% by 2050 [1,2,3].

Strategies for the treatment and eventual disposal of MSW have been developed with the aim of limiting its environmental impact [4,5]. Initially, the composition of municipal waste was deeply investigated in different countries [6,7,8,9,10,11,12,13,14]. To promote the recovery and recycling of materials from urban waste, residential curbside recycling programs have been adopted in several countries [15] and technologies suitable for sorting homogeneous waste fractions have been developed. In the case of separate collection, the citizens separate their recyclables into dedicated containers placed at the curbside. In this manner, fractions constituted, for instance, by plastics or paper only can be collected separately from the other waste (organic fraction of MSW). The different kinds of plastic can be further sorted, exploiting different techniques [3]. In a different approach, mixed waste is collected from households and then sorted at central facilities [15].

The implementation of separate collection systems and sorting processes has greatly promoted the recycling of waste components. Plastics, mainly polyethylene (PE), polypropylene (PP) and polyethylene terephthalate (PET), constitute a significant fraction of MSW that can be recycled. Different methods for the recycling of plastics recovered from municipal solid waste have been developed. Plastics can be subjected to mechanical recycling, but this process can, in principle, only be performed on single-polymer plastic [16]. After separation of a single kind of polymer and washing to remove contaminants, the material is extruded and pelletized. The pellets can be used as feedstock for manufacturing new goods. Alternatively, chemical recycling can be adopted to convert plastics into smaller molecules to be used as a feedstock to produce new polymeric materials.

The valorization of comingled waste (such as the organic fraction of MSW) is more challenging. However, mixed waste containing organic materials and plastics can also be valorized. For instance, they can be subjected to pyrolysis, resulting in the production of fuels [6,7,17]. Energy can be recovered by burning mixed waste or co-recycling household waste and green waste [18]. Residual or mixed MSW is frequently submitted to Mechanical–Biological Treatment (MBT), based on the combination of mechanical treatment and aerobic or anaerobic decomposition. The MBT can be designed for the production of refused derived fuel (to be utilized in waste to energy plants or as secondary solid fuel for clinker production), biogas and stabilized organic compost [15,19].

The Life Cycle Analysis (LCA) approach [17,20] was used to compare the different options for waste disposal in terms of environmental and human health impact, costs, and benefits. A hierarchy was therefore established. Recycling was found to be the best solution, while landfilling was the worst one. For instance, it was estimated that the recycling of plastic pellets requires only 5–7% of the energy needed for producing pellets starting from virgin materials [20]. In accordance with these results, the European Commission policy strongly promotes recycling of MSW, which should increase to 70% of produced waste by 2030, and it is committed to phasing out landfilling of non-treated MSW [15].

Despite the efforts spent promoting separate collection, recyclable materials are still present in residual and organic waste produced in households, which contains organic materials (e.g., leftovers, fruit peel, discarded parts of vegetable) mixed with recyclable plastics (such as shoppers and food packaging). In some European countries (such as Italy), the use of non-compostable plastics for shopper production has been banned, and, nowadays, bioplastic-made shoppers are allowed only. Biodegradable polymers proved to be environmentally friendly, as they, in similar manner to organic food waste, can be degraded to organic fertilizer. However, a mixture of biodegradable polymers and non-compostable ones still can be found in residual domestic waste (leftover waste in systems with separate collection) and constitutes 5–10% of the waste. Recovery and recycling of non-compostable plastic from this residual mixed waste is very challenging because of the complexity of the mixture composition and the presence of several contaminants in post-consumer plastics [10]. The possible origin of undesired substances in recycled plastics is polymer and additive breakdown products as well as contamination from external sources. Recycling could involve the migration of potentially hazardous substances from the recovered plastics to new goods. Research has been addressed to identify non-intentionally added substances in recycled plastics and to find possible solutions [10,21,22]. Narrowing the variety of additives used in plastic manufacturing and developing more efficient washing units for the removal of dirtiness have been proposed as countermeasures.

The present paper deals with the recovery of plastic fractions from waste rejected by the plant of the GAIA SpA company (situated in San Damiano d’Asti, Italy) designed to produce compost starting from the organic fraction of municipal solid waste (OFMSW). This rejected fraction would otherwise be sent to landfill after aerobic stabilization. The present work aimed at developing the recovery process, investigating the characteristics of the recovered polymers, and evaluating their suitability for manufacturing new plastic goods.

## 2. Materials and Methods

### 2.1. Waste Recovery at the Composting Plant

A discarded waste fraction resulting from OFMSW treatments was carried out in the plant of the GAIA company (San Damiano d’Asti, Italy). The GAIA unit is designed to up to 90,000 tons of OFMSW (more precisely, a mixture of OFMSW and agricultural waste generated from mowing and pruning) every year to produce compost. Firstly, the material to be treated is grinded in a dedicated machine. Afterwards, it is submitted to the process of aerobic digestion for 40–50 days.

The process of aerobic digestion starts with the preparation of the feedstock mixture. Once the mixture has been created, it is inserted inside the biocells, where the termo-hygrometric conditions are precisely set and maintained. The air inflow (10,000 m^3^/h per biocell) must be precisely regulated and maintained; the material matrix must be permeable to maintain the aerobicity. The process of maturation is usually divided into two phases (“Accelerated Maturation” and “Slow Maturation”), for a total duration of approximately 40/50 days. During the Accelerated Maturation, the humidity must be set between 35% and 60%, the O_2_ concentration must remain above 5%, and the temperature must remain above 55 °C for at least three days. The same thermo-hygrometric conditions must be ensured in the subsequent Slow Maturation Phase, except for the humidity, which must remain within the 30–55% range. At the end of the biocell process, the resulting compost is still rich in contaminants; therefore, to increase its quality, it is necessary to perform accurate sieving. In this manner, mixed waste containing non-biodegradable plastic, metals, and organic contaminants is separated from the compost. The sieving machine consists of two subsequent rotating drums equipped with holes. The sieving process creates three flows: quality compost (<9 mm in size, agricultural grade fertilizer), waste (mainly plastic residues of intermediate size, between 9 mm and 50 mm), and greater size residues (mainly wood to be recirculated, >50 mm in size). The last phase of the process applies to the first sieved flow and consists of the final maturation: 30 additional days of simple aeration of the pile. The sample for the experimentation was extracted from the waste residues of the intermediate dimension, which represents 10% of the waste treated in the plant.

### 2.2. Sorting of Plastic Fractions

Plastic fractions were sorted from a batch of 1.5 tons of the discarded waste using a new-generation NIR separator (Entsorga, Tortona, Italy) temporarily located inside the GAIA treatment plant. To this end, the waste was placed on a conveyor belt, where its plastic components were identified by a hyperspectral camera and blown away by a compressed air ejection system that sent the different plastics into separate baskets. Several polymers can be identified by the hyperspectral camera when they are subjected to light radiation, since they reflect a radiation spectrum that is the fingerprint of each specific polymer. The batch was sorted by the NIR separator according to several subsequent steps: separation of polyvinyl chloride (PVC), polyethylene (PE), polypropylene (PP), and polyethylene terephthalate (PET). In this manner, 153.8 kg of PE, 15.7 kg of PP, 14 kg of PET, and 6.7 kg of PVC were sorted. The single plastic fractions, excluding the PVC one, were homogenized by mechanical grinding, and representative samples of 1.5 kg were taken from each fraction for further investigation. Firstly, these samples were heated at 100 °C for 4 h and then weighted again to measure their initial moisture content. The samples were then washed and dried in the laboratory to remove non-polymeric organic and inorganic contaminants. To this end, the samples were dispersed in water under mechanical stirring and the plastics were removed from the dispersion. PE and PP were separated by flotation, since they are lighter than water. PET was separated from the liquid and from small particles of contaminants by sieving the dispersion through a 5 mm sieve. This washing and retrieving process was repeated several times on each sample containing plastics, and finally, the recovered plastic fraction was dried at 100 °C for 4 h and weighted. The difference in weight measured before and after the washing and drying procedure allowed us to assess the quantity of contaminants. Three batches of plastics, nominally made of PE (850 g), PP (850 g), and PET (1000 g), were recovered after the washing and drying process. The solid contaminants were also collected after the plastic removal by filtering the water dispersion. This residue was analyzed to assess its composition.

### 2.3. Characterization of Separated Fractions

The three plastic batches were characterized using Fourier Transformed Infrared Spectroscopy (FT-IR), X-ray diffraction (XRD), Differential Scanning Calorimetry (DSC) and Thermal Gravimetric Analysis (TGA). Infrared spectra were obtained in the range 500–4000 cm^−1^ using a Perkin-Elmer FT-IR Frontier spectroscope with a resolution of 1 cm^−1^. X-ray diffraction was carried out using a Malvern Panalytical X’PERT PRO PW3040/60 diffractometer with Cu Kα radiation at 40 kV and 40 mA. A Perkin Elmer Pyris 1 instrument was used for DSC tests. The samples were heated from 50 °C to 300 °C under nitrogen atmosphere (gas flow 30 mL/min, heating rate 10 °C/min) and then cooled; heating and cooling curves were recorded. Thermal gravimetric analyses were conducted with the Mettler Toledo TGA/SDTA851 equipment both in inert atmosphere (argon flow; 50 mL/min) and oxidizing atmosphere (air flow; 50 mL/min), heating the samples from 25 °C to 750 °C with a heating rate of 10 °C/min. Similar analyses were performed on the residue constituted by the contaminants with the aim of assessing the effectiveness and the selectivity of the washing process.

### 2.4. Processing Recycled Tensile Specimens and Testing

The three batches of plastic were processed to obtain pellets. Pellets of PE, PP, and PET were produced using a co-rotating twin screw extruder LEISTRITZ ZSE 18/40D with the following characteristics: diameter Φ = 18 mm and L/D ratio = 40. The extrusion flow rate was maintained at 3 kg/h. The barrel temperature was set at 190 °C for PE and PP, at 400 rpm with a spinning pressure of 20 bar, while PET was processed at 245 °C with a pressure of 40 bar.

Specimens with “dog bone” shape for mechanical tests (75 × 4 × 2 mm^3^) were obtained using a Babyplast 610P Standard injection molding machine, from CRONOPLAST SL (Abrera, Spain), operating at 190 °C for PE and PP and at 240 °C for PET. A first injection time of 20 s at 90 bars for filling the mold and a second injection time of 20 s at 80 bars of maintenance were adopted.

An MTS Criterion Model 43 dynamometer (with 5 kN load cell) equipped with a contact extensometer was used for tensile tests. A strain rate of 1 mm/min was adopted for the measurement of the elastic modulus, while the stress/strain curves were recorded with a strain rate of 10 mm/min. Seven specimens for each kind of material were tested according to standard UNI EN ISO 527-1,-2. The microstructure of the molded specimens was observed, after cutting, on their polished section using a Leica DMI 5000 M optical microscope. The density of both pellets and injection molded specimens was measured for each recovered plastic fraction using the Ultrapyc 5000 gas pycnometer from Anton Paar Quanta Tec. Inc. (Boyton Beach, FL, USA).

## 3. Results and Discussion

### 3.1. Composition of the Fraction Discarded by the Composting Plant

This fraction, separated by sieving the waste that was treated to produce compost, showed very complex composition because it contained several different plastics, metal fragments, and multilayer/multicomponent films originally used for packaging. The FT-IR analysis performed on several fragments sampled from this mixture showed that the main polymeric components of this waste were PE, PP, and PET; other minor components were polybutylene terephthalate (PBT), polyurethane (PU), acrylonitrile butadiene styrene (ABS), and polyamide (PA). There was no evidence of the presence of bioplastics (such as polybutylene adipate-co-terephthalate); reasonably, this kind of plastic underwent complete decomposition during the aerobic digestion of the waste, conducted under an oxidizing atmosphere before the sieving step.

### 3.2. Composition of the Batches Recovered by the NIR Separator

The content of plastic, moisture, and contaminants in each batch sorted by the NIR separator is shown in Table 1. These batches mainly contained plastics (58% wt. of PE, 57% wt. of PP and 68% wt. of PET, respectively), from 6% wt. to 13% wt. of moisture, and a non-negligible amounts of organic and inorganic solid contaminants. It can be inferred that the main part of the organic impurities was contained inside the MSW, while additional contaminants formed during the treatment of bio-oxidation performed on the original waste before the separation of the discarded fraction via sieving. The organic particles and moisture formed a slurry that coated the surface of the plastic fragments and had to be removed before the plastic recycling.

### 3.3. Effectiveness of the Washing Step

**Figure 1:**
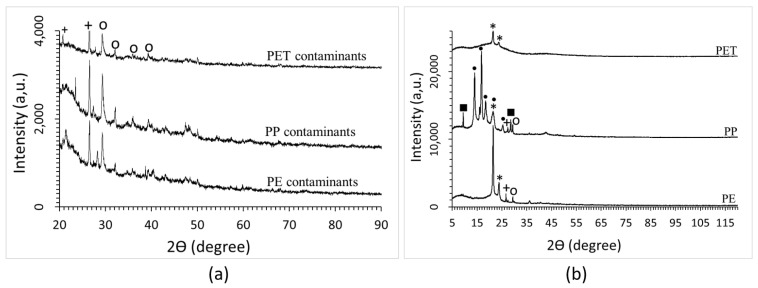
XRD of contaminants (**a**) and polymeric fractions (**b**): o = CaCO_3_, ■ = Mg_3_Si_4_O_10_(OH)_2_, + = SiO_2_, * = HDPE, • = PP.

The washing step allowed us to take away most of the impurities that contaminated the plastic fractions sorted by the NIR separator. The XRD spectrum of the solid impurities removed by washing is depicted in Figure 1a. From the broad shape of the XRD curves in the 2θ range between 20° and 50°, it can be inferred that most of this material is amorphous; however, sharp peaks due to silica and calcium carbonate can also be appreciated in the XRD patterns. On the other hand, there is no evidence of the presence of crystalline polymers in the XRD spectra of solid contaminants separated from the plastic batches (Figure 1a).

Differently, the XRD analysis of the plastic fractions after cleaning (Figure 1b), and the comparison with literature data [23,24,25,26,27,28], shows that each plastic batch mainly contains polymers. The XRD spectrum of PE shows two sharp peaks attributable to polyethylene (HDPE) placed at 21.6° (110) and 23.9° (200). The characteristic peaks of PP placed at 14.19° (110), 17.05° (040), 18.65° (130), 21.36° (111), 21.88° (−131), and 25.70° (060) can be clearly distinguished in the XRD of the plastic fraction selected as PP by the NIR separator. In the spectrum of the PET fraction, only two rather weak peaks at around 21.6° and 24° can be distinguished. They can be attributed to the PE (110) and (200) peaks. Several important peaks of crystalline PET, and in particular the (101) peak placed at 25.9°, are missing instead. This feature suggests that amorphous PET was collected by the NIR equipment.

The batch sorted as PP likely also contains high-density PE, as in its spectrum, the PE (200) peak (overlapped to (111) peak of PP) can be observed. Some signals attributable to inorganic materials (such as CaCO_3_, SiO_2_, and talc) can be detected in the XRD of PP fraction, while the PE fraction is contaminated by SiO_2_ and CaCO_3_. Talc peaks were observed at 9.5° (002) and 28.7° (006). Talc very likely was formerly present in the recovered plastic goods, since this material is currently used as a filler in the formulation of commercial plastics based on PP. In this case, talc is strictly mixed with the polymer and cannot be taken away by washing.

The presence of CaCO_3_ and SiO_2_ can be ascribed to incomplete effectiveness of the washing process carried out by exploiting basic laboratory facilities; this washing step could be improved when cleaning the plastics in industrial facilities.

**Figure 2:**
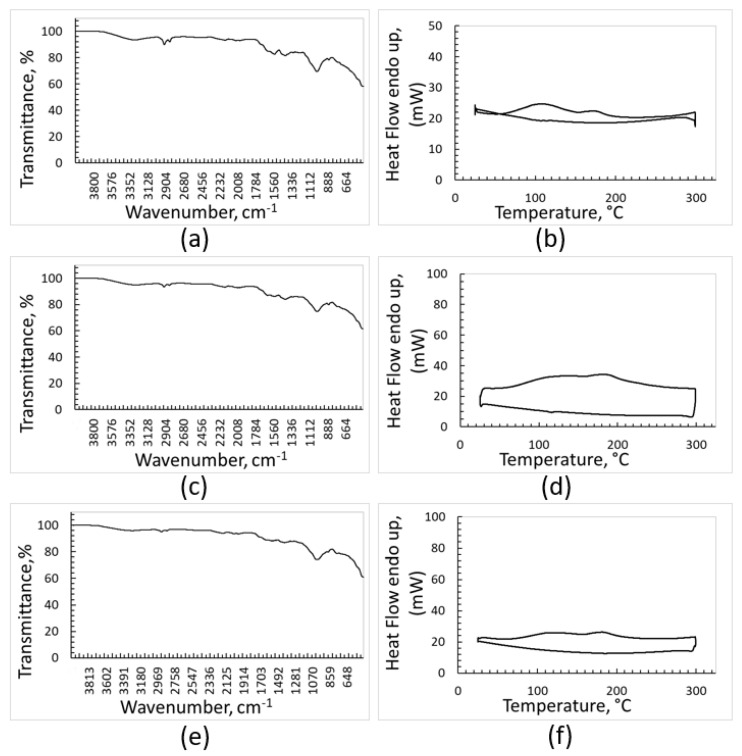
FTIR and DSC analyses of contaminants removed from: PE (**a**,**b**), PP (**c**,**d**), PET (**e**,**f**).

**Figure 3:**
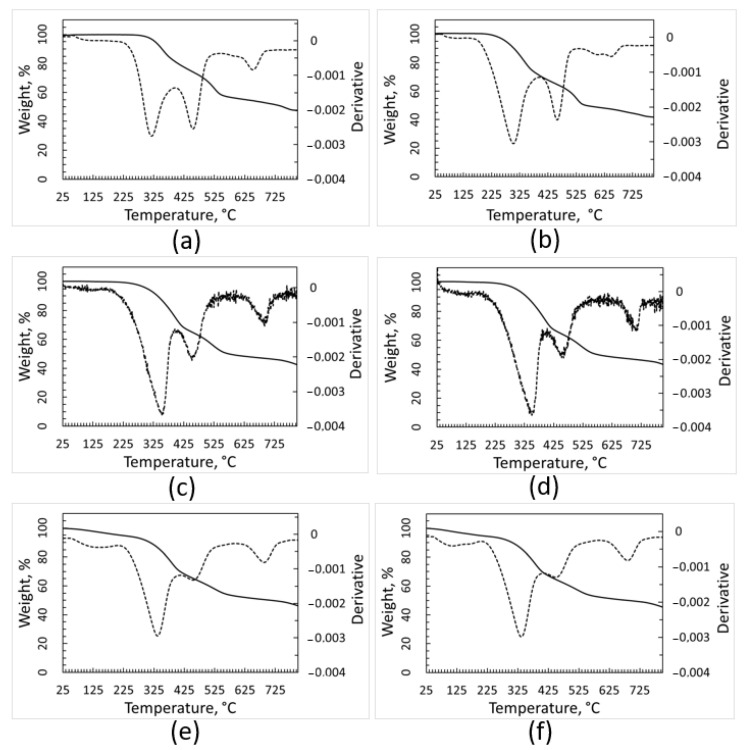
TGA and DTGA (dotted line) curves of contaminants removed from plastic batches. Curves recorded under inert atmosphere: PE (**a**), PP (**c**) and PET (**e**). Curves recorded under air, PE (**b**), PP (**d**), and PET (**f**).

The FT-IR, DSC, and TGA analyses of the removed contaminants gave more information about their composition and confirmed the efficacy of the cleaning treatment for plastic separation (Figure 2 and Figure 3). The FT-IR spectra of the solid contaminants separated by washing from the samples do not show signals of significant intensity attributable to crystalline polymers (Figure 2). However, very weak signals can be seen at around 2900 cm^−1^ in the spectra recorded for the contaminants removed from PE, PP, and PET plastic fractions, and they can be attributed to traces of PE or PP.

Likewise, melting peaks of crystalline polymers cannot be clearly appreciated in the DSC curves of the solid impurities (Figure 2). On the other hand, the DSC heating curves (upper curves) are not completely flat, as they show some deviations at around 120 °C and 170 °C, where melting of crystalline PE and PP, respectively, occurs. This feature strengthens the FT-IR outcomes, confirming that almost all polymeric materials are efficiently recovered when washing the plastic fractions sorted by the optical separator.

The TGA curves (Figure 3) of the pollutants separated from the plastic fractions show a complex trend, suggesting that this residue consists of several different substances. These curves are very similar for the contaminants of the different plastic fractions. The degradation phenomena occurring when TGA was performed under inert atmosphere (such as thermal decomposition) or when the analysis was performed under air (which causes oxidation, too) resulted in very similar weight decrease (of about 60%). This outcome seems consistent with the presence of significant amounts of calcium carbonate within the contaminants, as its thermal decomposition gives rise to a 44% weight decrease. A solid residue representing about 40% of the initial sample weight was left at the end of all the TGA runs up to 750 °C, showing that inorganic matter constituted a significant fraction of the contaminants.

All the results of the FT-IR, DSC, and TGA analyses concur to demonstrate that the impurities can be removed from the samples sorted by the optical separator without significant loss of the plastic components.

### 3.4. Characterization of the Cleaned PE Batch

**Figure 4:**
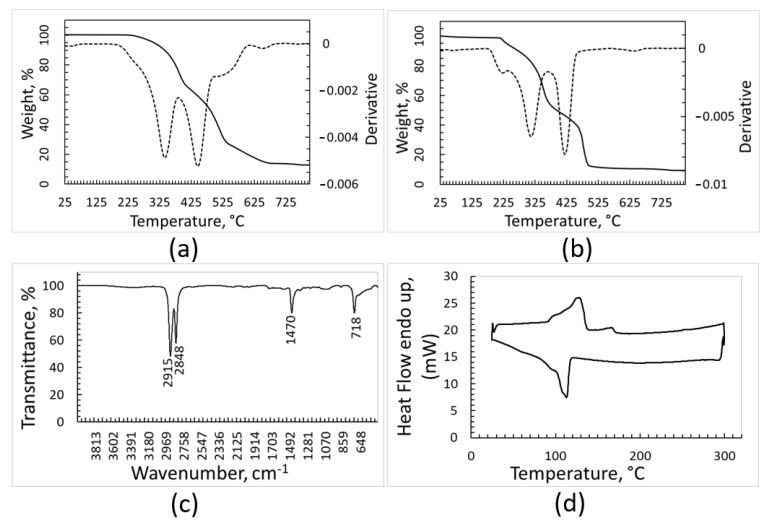
Characterization of the PE fraction: TGA and DTGA (dotted line) curves recorded under argon (**a**), TGA and DTGA recorded under air (**b**), FT-IR spectrum (**c**), DSC heating and cooling curves (upper and lower curve, respectively) recorded under nitrogen atmosphere (**d**).

The TGA curves, FT-IR spectrum and DSC traces of the batch identified by the optical sorter as PE are shown in Figure 4. The thermal gravimetric analysis suggests the presence inside the sample of materials with different thermal stability and oxidative behavior (Figure 4a,b). When the heating run is carried out under argon (Figure 4a), the material decomposition starts at around 225 °C and occurs according to an irregular trend with the temperature increase; a residue corresponding to about 10% of the initial sample weight is left after the thermal degradation. According to the literature, the thermal degradation of PE during TGA test occurs in a single step [29,30]. In the TGA curve of Figure 4a, the stronger peak at 448 °C fits with thermal degradation of high-density polyethylene (HDPE) [29], while the degradation of low-density polyethylene (LDPE) is expected to be placed at about 475 °C [30]. In addition, according to [31], the DTGA curve of recycled HDPE shows two peaks at 350 °C and 413 °C due to degradation of the material that occurred before recycling. Additionally, the DTGA curve of recovered PE fraction (Figure 4a) shows the presence of these two peaks in the region reported in the literature.

The TGA analysis performed under oxidant atmosphere (Figure 4b) further strengthens the hypothesis that different kinds of polymers, and impurities as well, are present inside the sample, as the curve of weight loss is structured and its derivative shows three maximum points. The first phenomenon with onset at around 175 °C may be due to oxidation of organic contaminants, which is consistent with the literature reporting that organic carbon contained in the soil undergoes oxidation starting from about 180 °C [32]. The two subsequent very sharp peaks in the DTGA curve can be attributed to oxidation of polymers with different microstructures. Additionally, in this last environmental condition, the solid residue after oxidation/degradation amounts to 10% of the initial weight. The solid residue of 10% wt. resulting from TGA analyses is not surprising, as inorganic components should suffer neither decomposition nor combustion and the burning or thermal decomposition of polymers typically leaves a carbonaceous solid residue. In addition, polymers are frequently charged with inorganic fillers for practical applications.

In the FT-IR spectrum (Figure 4c), the intense peaks characteristic of crystalline PE, placed at 2915 cm^−1^, 2848 cm^−1^, 1470 cm^−1^, and 718 cm^−1^, can be clearly distinguished, while other signals of significant intensity cannot be seen. Nonetheless, it should be considered that some strong peaks of PP are in the range between 2830 cm^−1^ and 2950 cm^−1^, and therefore could be overlapped with those attributed to PE. The IR spectroscopy confirmed that most of the batch was constituted by PE, possibly mixed with PP.

The DSC analysis (Figure 4d) seems to support this hypothesis, since the heating curve shows not only overlapped endothermic effects in the range of 107–130 °C, where polyethylene with different crystallinity degrees is expected to melt, but also a weak endothermic effect at about 163 °C, which is the melting temperature of PP. The cooling curve shows a sharp exothermic effect with a shoulder at a lower temperature, and therefore it is consistent with the crystallization of two polymeric species.

### 3.5. Characterization of the Cleaned PP Batch

**Figure 5:**
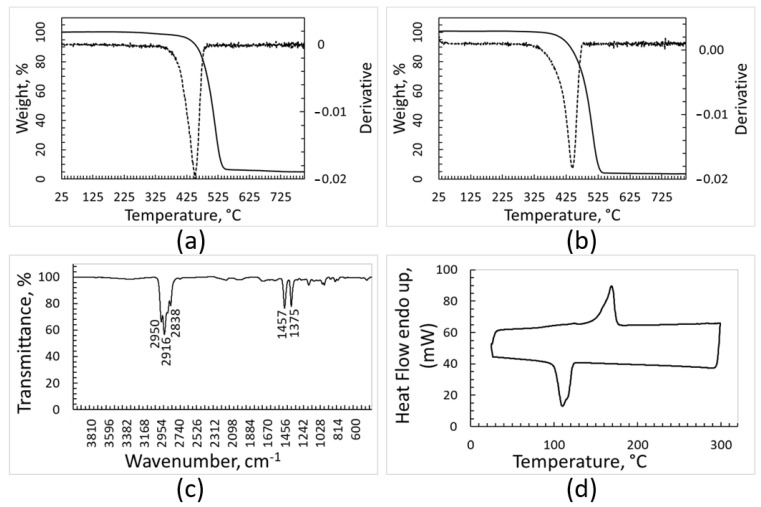
Characterization of the PP fraction: TGA and DTGA (dotted line) curves recorded under argon (**a**), TGA and DTGA recorded under air (**b**), FT-IR spectrum (**c**), DSC heating and cooling curves (upper and lower curve, respectively) recorded under nitrogen atmosphere (**d**).

The two TGA analyses, performed under argon and air, respectively (Figure 5a,b), evidence progressive weight loss without any shoulder or trend irregularity. The derivative curves show a single peak. The position of the peak in the DTGA trace is well consistent with the DTGA curve for PP reported in some literature [33]. It should be considered that deviation from this position has been also reported [34], and that a shift towards lower temperatures was observed for PP waste and PP samples after several reprocessing cycles or oxidative decomposition [34,35]. The residual weight after thermal decomposition under inert atmosphere is 7% of the initial sample weight, while the residue after oxidation represents 2% of the initial weight. These features are consistent with a sample mainly constituted of a single polymer. The FT-IR spectrum of PP batch (Figure 5c) shows several peaks partially overlapping in the range between 2838 cm^−1^ and 2950 cm^−1^. The peaks placed at 2838 cm^−1^, 2916 cm^−1^, and 2950 cm^−1^ are characteristic of PP, even though PE also presents peaks in this region (namely at 2915 cm^−1^ and 2847 cm^−1^). The two strong signals at 1375 cm^−1^ and 1457 cm^−1^ confirm that PP is the main component of this batch. The lack of the peak of PE at 1470 cm^−1^ and the peak of PET at 1714 cm^−1^, in spite the fact that they are very strong in the IR spectra characteristic of PE and PET, respectively, suggests that the possible contamination of the PP sample with other plastic is limited. The results of calorimetric analyses are shown in Figure 5d. The DSC analysis shows only the endothermic phenomenon of PP melting with a maximum at 160 °C and a rather sharp exothermal crystallization peak occurring during cooling at about 110 °C. These thermal and spectroscopic analyses suggest that the batch sorted by the NIR apparatus as PP is basically constituted by this polymer.

### 3.6. Characterization of the Cleaned PET Batch

**Figure 6:**
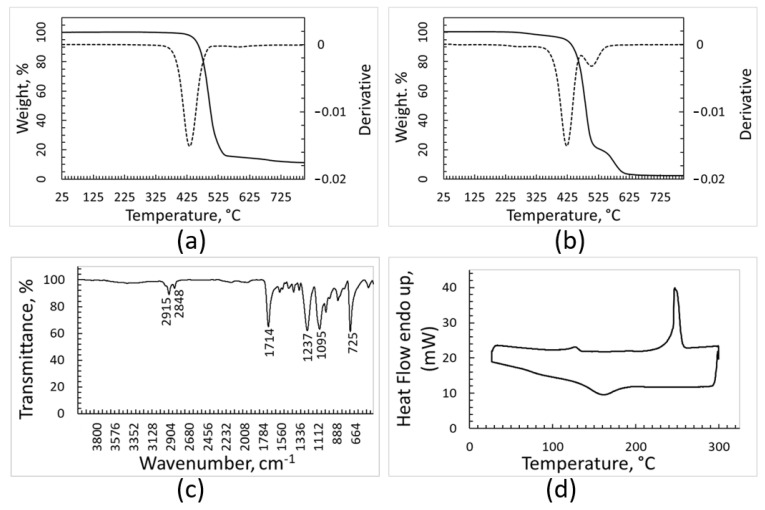
Characterization of the PET fraction: TGA and DTGA (dotted line) curves recorded under argon (**a**), TGA and DTGA recorded under air (**b**), FT-IR spectrum (**c**), DSC heating and cooling curves (upper and lower curve, respectively) recorded under nitrogen atmosphere (**d**).

The results of the TGA analysis (Figure 6a,b) are different when the analysis is carried out in an inert or oxidizing atmosphere. The TGA curve recorded in an inert atmosphere shows a progressive and regular weight decrease with the temperature increase, starting from 350 °C. The peak at 435 °C in the DTGA curve matches with the results obtained for PET thermal gravimetric analysis performed under the same experimental conditions [36]. The TGA curve of the PET batch recorded under air is more structured, showing a main oxidation phenomenon with a maximum in the DTGA curve at 425 °C and a second oxidation process occurring above 500 °C. The TGA curve of the PET sample recorded in air shows that it undergoes oxidation in a very different manner with respect to PE and PP samples, because the oxidation of these last two samples is completed below 500 °C, while in the case of the PET curve, the end of oxidation happens more than 100 °C later. The first peak in the derivative of the TGA curve seems compatible with the oxidation of PET [36], while the second one could be attributed to degradation of impurities. The residue left after the TGA run is equal to 10% of the initial weight when the analysis is performed under inert atmosphere, but close to zero (about 1% only) when it is performed under air.

Figure 6c reports the FT-IR spectrum of the PET sample, which looks very complex. In fact, in addition to the peaks placed at 725 cm^−1^, 1095 cm^−1^, 1237 cm^−1^ and 1714 cm^−1^ that belong to PET, other peaks of non-negligible intensity can be also found. The clear signals placed in the range between 2830 cm^−1^ and 2950 cm^−1^ could reasonably be attributed to PE and/or PP, and the spectrum shows several additional weak peaks below 1700 cm^−1^.

From the FT-IR spectrum, it can be concluded that the PET batch is mainly contaminated by other polymers. The results of DSC analysis (Figure 6d) strengthen this hypothesis. The heating curve detected by DSC shows a more important endothermic phenomenon at 250 °C, which is consistent with the melting of PET. The feeble peak at around 130 °C can be due to the presence of PE in the sample.

### 3.7. Microstructure and Tensile Properties of Recycled Polymeric Samples

The density of the samples produced by pelletizing and injection molding the plastic fractions recovered from the OFMSW is compared in Table 2 with the density values expected for specimens made of pure PE, PP, and PET. In this table, the density values measured on the pellets used for the molding process are also reported. The densities of the PE, PP, and PET molded polymers, as well as their other physical and mechanical properties, greatly depend on the crystalline degree of these materials, and therefore the density values can vary within a more or less wide range. The amorphous zones of PE should display density of 0.86 g/cm^3^, while the density expected for the crystalline areas is higher and closer to 1 g/cm^3^. In the case of PET and PP, the density also can change within a range, but this range is narrower for PP. Table 2 shows that the density measured on the pellets produced by using the recovered polymeric fractions was systematically lower than that of the printed samples owing to a certain degree of porosity that was eliminated, or at least greatly reduced, by injection molding.

The density of the printed samples mainly containing PE and PP was a bit higher than expected because of the presence of inorganic species that contaminated the plastic and were identified by XRD analysis. On the contrary, the density of the amorphous PET fraction was lowered by PE and PP, whose presence was assessed by XRD, FT-IR, and DSC analyses.

**Figure 7:**
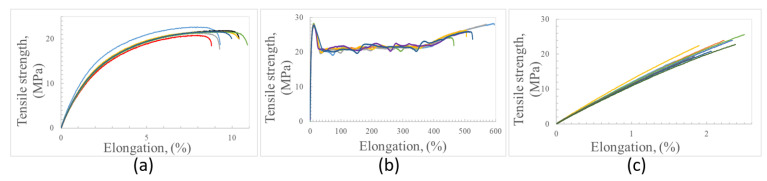
Tensile stress–strain curves for recycled PE (**a**), PP (**b**) and PET (**c**).

The stress–strain curves resulting from the tensile test of PE, PP, and PET samples produced by using 100% of recovered plastic fractions are compared in Figure 7. These curves show very different trends depending on the kind of polymer. Polyethylene at the beginning suffers elastic deformation, and afterwards, progressive plastic deformation till breakage. The elongation at the break of PE specimens was rather low. However, the presence of inorganic contaminants can cause a significant reduction in ductility.

The polypropylene tensile curves (Figure 7b) show initial elastic deformation, yielding, and then progressive important elongation before breaking (about 520%). Commercial PP products can show a very wide range of strain values at break, with the highest values reaching up to 750% [37]. The presence of talc in this recovered plastic does not seem to significantly limit the ductility probably because its percentage is rather low.

The polyethylene terephthalate tensile curves are characterized by an almost linear trend till the failure, which occurs a little after sample elongation. In addition, the XRD and FT-IR analyses showed that the PET-recovered fraction was a blend of PET with other polymers (mainly PE and PP). The elongation at break of PET goods can range in a wide field of values (from some percent to hundreds of percent), and several kinds of PET-based goods could be recovered by the optical sorting process.

The tensile characteristics of the samples made of recycled plastics are summarized in Table 3. A comparison of these experimental results with the tensile properties of virgin polymers and commercial products is quite hard because the recovered plastic fractions are, unavoidably, a mixture of commercial plastics showing different microstructures and containing different kinds of fillers and additives. However, some consideration can be made, as follows. The elastic modulus values calculated from the slope of the initial part of the stress–strain curves, and recorded using 1 mm/min strain rate, were very well reproducible for all the sets of polymeric samples under investigation. Values of elastic modulus of 1.397 GPa and 1.279 GPa were measured for recycled PE and PP, respectively. The tensile modulus of recycled PET was equal to 1.968 GPa. Low-density polyethylene (LDPE) currently shows a modulus lower than 1 GPa, while the stiffness of high-density polyethylene (HDPE) progressively increases with the crystalline degree up to 1.5 GPa. The tensile modulus measured on the recycled PE is consistent with the prevalent presence of PE with crystalline structure. The tensile modulus of recycled PP is just a bit lower than typical values for PP.

The stiffness of the recycled PET seems rather low in comparison with that of virgin PET, whose modulus currently is around 3 GPa. However, it should be considered that both FT-IR and DSC analyses of the plastic fraction recovered as PET showed that this material also contains non-negligible amounts of PE and PP.

The average values of tensile strength detected for PE and PP are consistent with specimens mainly composed of these polymers. Commercial low-density PE currently shows tensile strength between 8 MPa and 26 MPa, while the strength of HDPE can increase up to around 32 MPa. The value of tensile strength measured for recycled PE (21.8 MPa) is not surprising at all, since the recycled PE material is constituted of a blend of recovered commercial PE products showing different mechanical features. The tensile strength of commercial PP is generally a bit higher than 30 MPa, while the recycled PP showed a strength of 28 GPa; the presence of polymeric and inorganic contaminants inside the recovered PP fraction can well justify this mismatch.

Commercial PET products can show very different tensile strengths (even ranging between 22 MPa and 95 MPa) so that the tensile strength of the recycled PET fraction is placed at the lower limit of this range of strength values. Additionally, in this case, it should be considered that the recovered PET batch is not composed of a single polymer and that the properties of commercial PET are currently improved by using additives.

**Figure 8:**
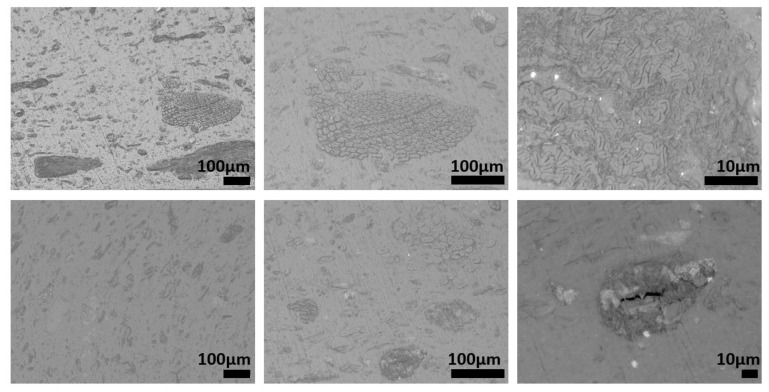
Microstructure of specimens produced using recovered PE.

**Figure 9:**
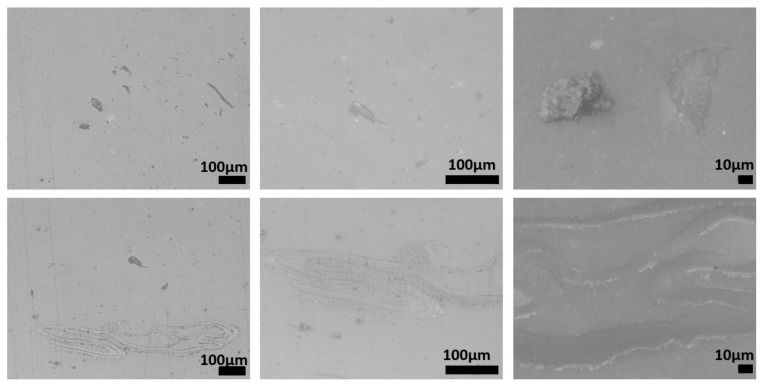
Microstructure of specimens produced using recovered PP.

**Figure 10:**
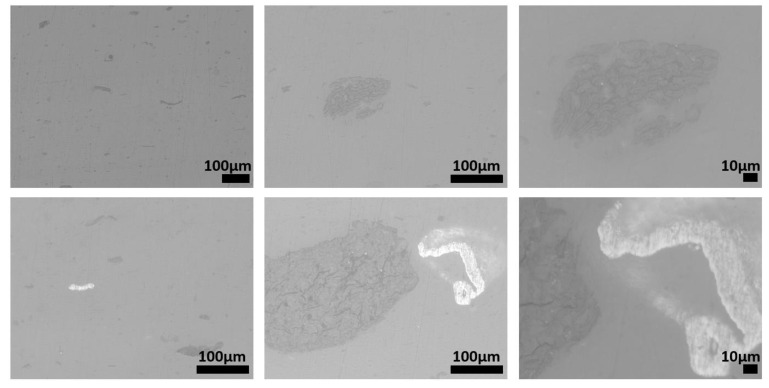
Microstructure of specimens produced by using recovered PET.

The microstructures of recycled samples of PE, PP, and PET are shown in Figure 8, Figure 9 and Figure 10, respectively. These figures show the specimens sections observed using an optical microscope at different magnifications. In each figure, the magnification increases, moving from the left to the right. All the pictures were taken to put in evidence as the microstructure of the specimens processed by injection molding of plastic fractions recovered from OFMSW is not homogeneous. Some particles and particle clusters can be seen in the microstructure of recovered materials, particularly in the case of PE and PET (Figure 8 and Figure 10). Microcracks can be found inside these particles, and they can probably promote the final breakage during tensile tests. Zones with different shade of gray and an elongated shape may be due to the presence of polymers different from the main one. The viscosity of these zones in the conditions adopted for injection molding is very likely different from that of the rest of the material, and therefore they are subjected to different deformation during the printing process.

The microstructures of the recovered polymers show different levels of homogeneity. The pictures taken at lower magnification show that the lack of homogeneity is higher in the case of PE (Figure 8) with respect to that of PP and PET (Figure 9 and Figure 10). However, it should be considered that the optical sorter is not able to distinguish between PE with different crystallinity degrees (namely HDPE and LDPE), and if this is the case, they are recovered in the same plastic fraction.

Despite the lack of homogeneity in the composition of the recovered plastic fractions, it was possible to find injection molding parameters suitable for producing items with tensile behavior similar to that of virgin polymers.

## 4. Conclusions

A processing path suitable for the recovery of polymers from the discarded waste of composting plants, currently landfilled, was investigated in this work. According to this process, about 640 tons of plastics (PE, PP and PET) could be recovered every year in the composting plant that furnished the samples of rejected waste used in this study. A NIR separator proved to be able to recover plastics from the composting plant waste, which shows very high content of moisture, organic particles, and inorganic impurities, and contains metal fragments and multi-component objects. However, moisture and organic materials strongly contaminated the surface of the plastic fragments; in addition, they formed a slurry which acted as a glue that bound fragments of different plastics together. For this reason, each batch of sorted plastic was contaminated with other polymers, and with water and organic material as well. The efficiency and the selectivity of the sorting process can very likely be greatly improved if the starting mixed waste is washed and dried before the NIR sorting treatment, thus taking the slurry from the surface of the plastic fragments. This cleaning step was adopted in the present investigation to make the recovered materials suitable for producing objects. PE and PP were quite easily separated from the contaminants by flotation, as the density of these polymers is lower than that of water. However, the washing of the PET sample was not so effective. Polyethylene was found to be the main contaminant of the PP batch, and vice versa. These two materials also contaminated the PET batch. After the washing treatment, samples suitable for tensile tests were processed by pelletizing and injection molding. The samples made of PE and PP showed mechanical features (such as modulus and strength) similar to those characteristics of the corresponding virgin materials, despite the contamination with different kinds of polymers and inorganic matter. The strain at break was high for PP (524%), but much lower for PE and, in particular, for PET. The presence of residual contaminants resulted in a lack of homogeneity in the microstructure of the printed materials, and it was very likely responsible for the poor strength of recycled PET and low ductility of recycled PE and PET. Conclusively, at least the recovered PE and PP fractions seem suitable for exploitation in compounding with virgin materials and the manufacture of goods. Improvement of compositional and mechanical features of the plastic fractions recovered according to the investigated route would require enhancements in the cleaning step and optimization of the molding process, with the help of fillers and additives.

## Figures and Tables

**Table 1:** Weight percent of plastic, moisture and solid contaminants in each batch sorted by the NIR separator.

Polymeric Fractions	Selected Polymer	Organic and Inorganic Contaminants	Water
Polyethylene (PE)	58%	36%	6%
Polypropylene (PP)	57%	30%	13%
Polyethylene terephthalate (PET)	68%	24%	8%

**Table 2:** Density of recycled polymeric fractions.

Density (g/cm^3^)	PE	PP	PET
Theoretical	0.86–0.97	0.90–0.91	1.30–1.40
Pellets	1.00	0.89	1.19
Printed samples	1.11	0.93	1.24

**Table 3:** Tensile strength, modulus, and strain at break of recycled PE, PP and PET.

Recycled Polymers	Tensile Strength	Elastic Modulus	Strain at Break
	σ_max_ ± SD (MPa)	E ± SD (GPa)	ε ± SD (%)
Polyethylene	21.8 ± 0.6	1.397 ± 0.024	10.0 ± 0.9
Polypropylene	28.0 ± 0.3	1.279 ± 0.050	524.2 ± 44.6
Polyethylene terephthalate	22.1 ± 2.6	1.968 ± 0.109	2.1 ± 0.3

## Data Availability

All the data presented in this study are available in the present article.
